# How to conduct a refractive outcome audit to improve biometry: a step-by-step guide

**Published:** 2025-09-19

**Authors:** Samuel Hailemichael Henok, Mattan Arazi, Maureen Kiaraho

**Affiliations:** 1 MSc Public Health for Eye Care Candidate, London School of Hygiene & Tropical Medicine, UK.; 2 MSc Public Health for Eye Care Candidate, London School of Hygiene & Tropical Medicine, UK and Resident Physician, Sheba Medical Center, Ramat Gan, Israel.; 3 MSc Public Health for Eye Care Candidate, London School of Hygiene & Tropical Medicine, UK and Consultant Ophthalmologist: County Government of Kiambu, Kenya.

**Regular audits help surgical teams to avoid systematic errors and improve IOL selection, thereby ensuring better refractive outcomes for patients.**

**Figure:**
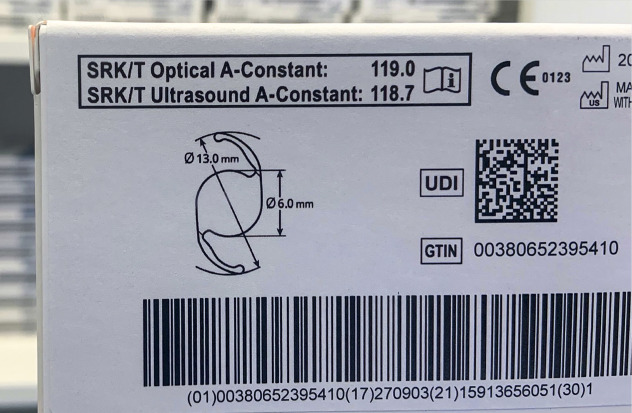
Applying the manufacturer's A constant without calibration can result in a refractive shift, often towards unintended hyperopic outcomes.

© JOHN BUCHAN CC BY-NC-SA 4.0

Postoperative refractive error is one of the most common causes of poor visual outcomes after cataract surgery, underscoring the need for more accurate biometry. Residual refractive errors after cataract surgery can significantly impair near, intermediate, and distance vision, with larger errors leading to worse visual outcomes and, ultimately, reducing quality of life.^1^

According to the United Kingdom's National Health Service benchmark guideline, 85% of eyes should be within 1.0 D and 55% within 0.5 D of the desired spherical equivalent (SE) refraction after cataract surgery.^2^ In countries where access to postoperative spectacles is limited, even small errors in biometry can limit patients’ ability to perform daily tasks.

Precise intraocular lens (IOL) power selection depends on various key elements, including:

- Accurate biometry measurements (axial length or keratometry readings)
- Accurate prediction of effective lens position
- Appropriate formula selection
- An optimised A constant that reflects local surgical techniques.

This article introduces the ABC refractive outcome audit framework to systematically address the above factors:

- **A (A-constant).** Optimising or calibrating the A-constant to take into account the effective lens position and other formula variables in order to ensure that it reflects local practice conditions
- **B (biometry).** Ensuring measurement accuracy across technicians and devices
- **C (customisation).** Aligning refractive outcome targets with patient needs and surgical realities.

## A. Optimising the A-constant

Accurate IOL power calculation relies on the precise calibration of the A-constant. The value of the A-constant depends on three factors: the IOL model, the biometry instruments used, and the desired position of the IOL implantation (either in the bag or in the sulcus).

Real-world surgical variations – including differences in surgical technique and variations in postoperative wound healing – can introduce systematic errors. Regular audits of refractive outcomes allow surgical teams to identify and correct these discrepancies, ensuring that the A-constant reflects local practice conditions.

An unoptimised A constant systematically skews outcomes towards hyperopia or myopia. Manufacturer-published constants are typically based on contact ultrasound biometry and may not account for the longer axial length measurements obtained with immersion or optical biometry. Consequently, applying these constants, without calibration, to modern optical biometry models can result in a refractive shift, often towards unintended hyperopic outcomes.^3^

The labelled constant on IOL packaging usually does not consider local surgical conditions. For new IOL introductions, it is advisable to adopt published constants from peer-reviewed sources until enough institutional data is gathered for optimisation. Constant optimisation recalibrates the formula's mean prediction error to zero, ensuring that the intended refractive target matches postoperative results. It has minimal effect on the dispersion of outcomes around the mean (i.e., the standard deviation); however, it increases the proportion of eyes falling within a particular target range.^4^ A study done at an eye department in the United Kingdom demonstrated that repeated optimisation of the A-constant increased the proportion of eyes achieving postoperative refraction within ±1.0 D of the target from 65% to 95%.^5^

### How to optimise the A-constant

1. **Collect postoperative refraction data**. For reliable results, record a minimum of 30 eyes consecutively, implanted with the same biometry device, IOL model, and surgical technique. (For the IOLMaster optical biometer, the recommendation is to use data from more than 50 eyes).^6^ Refraction should ideally be performed at 4 weeks postoperatively for phacoemulsification, at 4–6 weeks for manual small-incision cataract surgery, or after suture removal for extracapsular cataract extraction. Autorefractors may be a pragmatic alternative in outreach settings where manual refraction is unavailable, although you might want to verify the accuracy of the autorefraction by cross-checking through subjective refraction on selected patients.
2. **Calculate each patient's spherical equivalent (SE) prediction error.** This is the difference between the target SE and the actual postoperative SE. For example, if the target SE was −0.5 D and the achieved SE was +0.1 D, the error is +0.6 D (indicating hyperopic surprise).
$$\begin{array}{ccccc} \text{SE prediction error} & = & \text{Postoperative SE} & - & \text{Predicted}(\text{target})\text{SE} \end{array}$$
3. **Calculate the mean error.** This is defined as the arithmetic average of the prediction errors from a patient cohort:
  $$\begin{array}{ccc} \text{Mean error} & = & \frac{\text{Sum of prediction errors}}{\text{Number of patients}} \end{array}$$
  It shows how close the actual outcome aligns with the intended target in a group of patients and indicates the direction and magnitude of bias in your prediction.
4. **Adjust the A-constant.** A negative mean error suggests that the outcomes are generally more myopic than desired, while a positive mean error indicates a hyperopic tendency. If the mean error exceeds +0.3 D or –0.3 D, an adjustment to the A-constant is needed. If the mean error is positive (hyperopic outcomes), the A-constant should be increased by the magnitude of the error; if negative (myopic outcomes), it should be decreased by the magnitude of the error. The mean error should be as close to zero as possible after A-constant optimisation.
5. **After recalibration, repeat the audit annually to verify sustained accuracy.** Conduct audits sooner if there are changes to the IOL model or manufacturer, the biometry equipment or staff members, and the surgical technique.

## B. Ensuring accuracy in biometry measurements

Biometric measurements of the eye are necessary for accurate IOL power calculations, with axial length and corneal power being the minimum required parameters. Errors in axial length or corneal power readings – whether due to technician technique, equipment calibration, or patient factors – directly impact IOL power calculations. An error of 1 mm in axial length measurement can alter the IOL power by 2.5–3.0 D, while an error in corneal power of 1.0 D brings about an equivalent change in power of 1.0 D.^10^

Contact ultrasound methods introduce systematic errors in measuring axial length due to unintended corneal compression, with the magnitude of error influenced by the operator's experience. In contrast, immersion ultrasound techniques avoid corneal compression and can provide refractive results comparable to optical methods.^5^

### Steps to evaluate biometry measurement consistency

**Figure 1:**
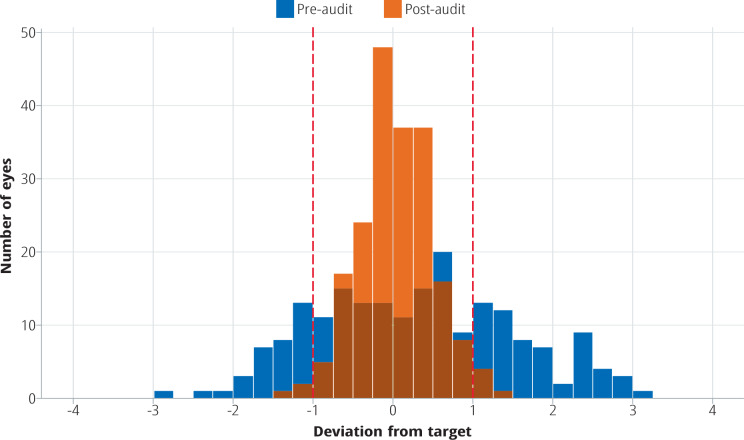
Distribution of refractive outcomes compared to the target before and after audit (with benchmark set of 85% of outcomes within ±1.0 Dioptre)

1. **Plot the distribution of refractive errors.** Generate a histogram of postoperative prediction errors (actual SE minus target SE) in 0.5 D increments. A well-calibrated and precise measurement should give a normal distribution centred on zero (Figure 1).

**Figure:**
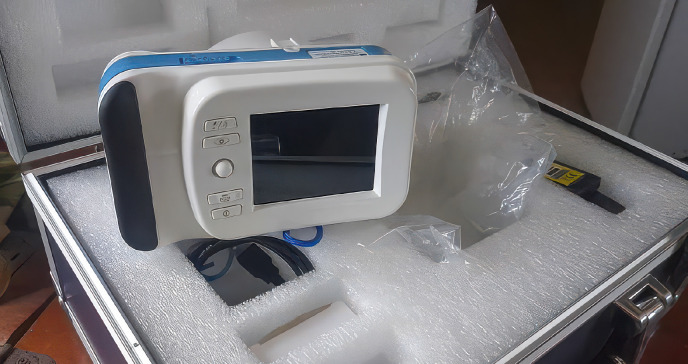
Tabletop autokeratometer (or autorefractor keratometer). The value of the A-constant depends on the equipment used. NIGERIA

© ANCILLA CATHOLIC EYE FOUNDATION CC BY-NC-SA 4.0

1. **Quantify outliers.** If the histogram is skewed to one side, consider adjusting the A-constant (Section A); if the spread of outcomes is wide with 15% or more outcomes >1.0 D from target, consider investigating inconsistencies in biometry measurement.^3^
2. **Compare measurements across technicians.** If the spread exceeds the set benchmarks, compare measurements across technicians. Ensure that at least three technicians measure the same subset of 10—15 patients (same eye, same session), recording axial length and keratometry values. Discrepancies exceeding 0.2 mm for axial length suggest technique-related errors, such as corneal compression during A-scan (resulting in artificially short axial lengths) or fluid bridge artefacts (causing overestimated axial lengths).^11^
3. **Standardise protocols.** Next, standardise biometry protocols to minimise variability. Repeat measurements to ensure consistency in the protocol. For A-scan users, prioritise immersion techniques or enforce consistent probe pressure in contact methods. Keratometry requires monthly calibration.
4. **Operator-specific A-constants (if needed).** If inter-technician variability persists, consider calculating operator-specific A-constants to account for individual measurement biases.

## C. Customise refractive targets to local needs: patient-centred outcomes

A one-size-fits-all target of emmetropia (0 D error) may leave patients struggling with daily tasks. Tailoring refractive outcomes to individual lifestyles and local realities improves quality of life and enhances patient satisfaction. While emmetropia provides sharp distance vision, presbyopia and the lack of a truly accommodative IOL require many patients to wear spectacles for near tasks. In low-resource settings, where postoperative access to spectacles is limited, and for patients accustomed to lifelong myopia, strict adherence to plano targets may diminish quality of life.

For example, a farmer in rural India may benefit from a deliberate myopic target of −1.0 D, which reduces dependence on spectacles for reading and improves the ability to perform critical daily tasks, like sorting seeds.

“Tailoring refractive outcomes to individual lifestyles and local realities improves quality of life and enhances patient satisfaction.”

When planning monovision – such as targeting −0.25 D in the dominant eye and −1.25 D in the non-dominant eye – anisometropia should not exceed 2.0 D; this will avoid intolerable imbalance.^11^

Preoperative counselling is essential. Simple questions like ‘How many hours a day do you spend cooking or reading?’, may reveal vision requirements and daily priorities. Hyperopia should be rigorously avoided, as even mild hyperopic refractive surprises are often poorly tolerated, particularly in regions with limited access to corrective spectacles. Patients must also be educated about the inevitability of spectacles for near vision if emmetropia is targeted, and they should be offered alternatives like affordable near vision spectacles.

## Conclusion

The ABC framework – A-constant refinement, biometry audits, and customised targets – provides a roadmap for conducting refractive outcome audits and improving cataract surgery outcomes in low- and middle-income countries. By integrating regular audits, standardised protocols, and patient-centred targets, surgical teams can reduce postoperative refractive errors and enhance patients’ quality of life.
